# Prevention of Lens Epithelial Cell Growth *In Vitro* Using Mibefradil-Containing PLGA Micro Particles

**DOI:** 10.2174/1874364100802010112

**Published:** 2008-06-12

**Authors:** Arne Weidmann, Sabine Kwittner, Ria Beck, Joachim Teller, Ludwig Jonas, J. Barbara Nebe

**Affiliations:** 1 Biomedical Research Centre, Cell Biology, University of Rostock, Schillingallee 69, D-18057 Rostock, Germany; 2 Dept. of Ophthalmology, University of Rostock, Doberaner Str. 140, D-18055 Rostock, Germany; 3 Micromod Partikeltechnologie GmbH, Friedrich-Barnewitz-Str. 4, D-18119 Rostock, Germany; 4 Electron Microscopic Centre, Medical Faculty, University of Rostock, Strempelstr. 14, D-18057 Rostock, Germany

**Keywords:** Calcium channel antagonist Mibefradil, lens epithelial cell adhesion, PLGA micro particles, organ culture model.

## Abstract

The prevention of the posterior capsule opacification is still unsolved. To interfere with proliferating cells the T-type calcium channel antagonist Mibefradil was immobilized in poly-lactic-co-glycolic-acid micro particles which were fixed at a capsular tension ring and tested in a human organ culture model as well as in human lens cells HLE-B3 **in vitro**. It is feasible to get a release significantly affecting cell viability and growth evaluated by MTT test and cell cycle analysis. In addition, Bionas^®^ sensor chips were used for time-dependent adhesion experiments in living lens cells. Interestingly, the concentration of Mibefradil which inhibited subconfluent cells is not effective in confluent cells. This is an important feature for the protection of the intact tissue in the eye.

## INTRODUCTION

Posterior capsule opacification (PCO) is still the major complication in cataract surgery in consequence of an activation of lens epithelial cells due to surgery [1-4] that involves cell phenotype changes [5], increased matrix production, proliferation and migration [6,7]. To date, most of the pharmacological drugs which have been therapeutically applied to block opacification [8] are limited in use due to serious damage of the surrounding tissues in the eye [9]. Interesting investigations concerning stress response of lens cells in the closed capsular bag **in vitro** were made by Duncan [10] using thapsigargin, hyperosmotic NaCl or distilled water as hyposmotic stress. The mechanism of action of thapsigargin is to disable calcium signalling by depleting the ER calcium store, thus leading to cell cycle arrest [10]. This is different from the cellular influence of the other tested solutions. Advances in the prevention of PCO have been made concerning the geometry of the intraocular lenses [11], thus sharp edged lenses were able to diminish the migration of lens cells [12, 13]. At least, this technique is not able to solve the long term problem of PCO [14].

The challenge of a suitable therapy to inhibit capsule opacification could be to specifically interfere with cellular adhesion mechanisms in outgrowing lens epithelial cells. The focus is also on the calcium signalling pathway. Our approach using the T-type calcium channel antagonist Mibefradil is based on the hypothesis that this drug could prevent cellular adhesion and function due to an inhibition of the calcium entry into the cell. An inhibition of the calcium entry was not only found for platelet cells [15] but we also provide evidence that our primary human lens epithelial cells in the cell culture express T-type calcium channels [16,17] as well as potassium channels [16-18], which were influenced by Mibefradil. Our previous studies demonstrated that cultured primary human lens epithelial cells which grew out from anterior capsules obtained from patients after cataract surgery detached from the tissue culture plastic due to incubation with Mibefradil. This was concentration dependent accompanied by structural changes of extracellular matrix proteins, fragmented actin cytoskeleton, and an altered organization of β1-integrin receptors as well as their reduced expression [16,18-20] as summarized in Fig. (**1**). All these findings suggest specific actions induced by Mibefradil. Our new studies were focused on (i) the inhibitory effect of Mibefradil especially on outgrowing cells (i.e. subconfluent), (ii) on a practicable approach to immobilize this drug in micro particles by modifying a solvent evaporation procedure with a constant release during a longer period, and (iii) to affix these particles on a capsular tension ring to test the inhibitory influence on the cell’s outgrowth in an organ culture model.

## MATERIALS AND METHODOLOGY

### Calcium Channel Antagonist and Immobilization Procedure

The T-type calcium channel blocker Mibefradil (1S,2S)-2-[2-[[3-(2-benzimidazolyl propyl] methyl amino] ethyl]-6-fluoro-1,2,3,4-tetrahydro-1-isopropyl-2-naphtyl-methoxy acetate dihydro-chloride) (Sigma) [7,22,15] was solubilized in distilled water (stock solution 50 mg/10 ml) for cell culture experiments. The stock solution was stored at 4 °C. There are no commercial relationships between Sigma and our department of cell biology.

Spherical PLGA (poly-lactic-co-glycolic-acid) micro particles were used for immobilization of Mibefradil dihydrochloride. Mibefradil containing micro particles were prepared by modifying a solvent evaporation procedure using ethyl acetate as dispersing solvent [23]. These micro capsules released the active agent with a daily average of 7.5 mg/l during a period of 50 days [24,25]. The micro particles were coupled on the PMMA (polymethyl methacrylate) surface of the capsular tension ring (Micromod Partikeltechnologie GmbH) [25] which contain 38 mg Mibefradil per gram. The total amount of Mibefradil on a capsular tension ring corresponds to a final concentration of 10, 20 und 30 µM after 24 h.

### Scanning Electron Microscopy

Cells cultured on cover slips were fixed with 4 % glutaraldehyde and dehydrated through a grade series of acetone. After critical point drying (Emitech K850, Emitech) and sputter-coating with gold (SCD 004, BAL-TEC), samples were examined using a scanning electron microscope DSM 960A (Carl Zeiss) at an accelerating voltage of 10 kV.

### Transmission Electron Microscopy

Samples were fixed with 4 % glutaraldehyde, postfixed in 1 % buffered osmiumtetraoxide (OsO_4_) followed by dehydration through a graduated series of acetone and embedded in epoxy resin araldite. Ultra-thin sections were prepared with an ultramicrotome (Ultratom III, LKB or Ultracut SWS, Leica), stained with uranyl acetate and citrate and then examined in the transmission electron microscope EM 902 (Carl Zeiss). Digital pictures were made by the CCD camera (Proscan) using the EFTEM software (Olympus Soft Imaging Solutions).

### Cell Culture of Human Lens Epithelial Cells

The immortalized human lens epithelial cells HLE-B3 (ATCC No: CRL-11421) were cultivated in tissue culture flasks (25 cm^2^, Greiner Bio-One) in Dulbecco’s modified Eagle medium (DMEM, Invitrogen) with 10 % fetal calf serum (FCS), 4500 mg/l glucose (high glucose), GlutaMAX, pyruvate and 1 % gentamicin (Gibco Invitrogen) at 37 °C and in a 5 % CO_2_ atmosphere.

### Organ Culture Model

The human phacocyst (capsular bag) was excised post mortem as recommended by Priglinger [26] and the lenticular cortex and nucleus was eliminated. This organ culture model should simulate conditions **in vivo** due to similar physiological circumstances. The phacocyst was cultivated in DMEM with 0.5 % FCS and 1 % gentamicin for 20 days before the pharmacologically modified micro particles were inserted and the Mibefradil released over 24 h.

### Cell Vitality Analysis

The inhibitory influence of Mibefradil-containing micro particles (concentrations 10, 20, 30 µM, after 24 h) was studied by measuring lens cell’s metabolism (methyl thiazolil tetracolium test, MTT test^®^, Roche). The principle of the test is an enzymatic cleavage of the tetracolium salt 3-(4,5-dimethylthiazol-2-yl)-2,5-diphenyltetrazoliumbromide (MTT) by active cells into water insoluble violet salt crystals. The spectrophotometric absorption was analyzed by an ELISA reader (Anthos 2010, Anthos Labtec Instruments) at 590 nm. The extinction is proportional to the metabolic activity of the cells. The MTT test was used for HLE-B3 lens cells as well as for the primary cells in the capsular bag as organ culture model. HLE-B3 cells were cultured in 24-well plates and incubated for 24 h with Mibefradil containing micro particles using release-concentrations of 10, 20, and 30 µM. Primary cells of the capsular bag were incubated with Mibefradil similarly.

The cell survival was studied by live/dead-viability test (Molecular Probes). HLE-B3 cells were cultured for 24 h in 6-well plates with tissue culture inserts (Thin Cert, pore size 0.4 µm, Greiner Bio-One) where Mibefradil containing micro particles were placed. Staining of the cells was performed according to the manufacturer’s instructions. Calcein stains living cells (green fluorescence) whereas ethidium homodimer indicates dead cells (red fluorescence). Light microscopy (Axiovert 25, Carl Zeiss) and laser scanning confocal microscopy (LSM 410, objective 40x/0.6, LD Acroplan ph2, Carl Zeiss) were used for the observations.

### Cell Cycle Analysis and Apoptosis

The HLE-B3 cells were cultured in 6-well plates. Mibefradil containing micro particles were placed on culture inserts (Thin Cert, pore size 0.4 µm, Greiner Bio-One) in these 6-well plates. The Mibefradil release corresponded to concentrations of 10, 20, and 30 µM. Incubation time was 24 h. For flow cytometry, HLE-B3 cells were suspended using 0.05 % trypsin/0.02 % EDTA solution (5 min at 37 °C). Then, cells were washed in phosphate buffered saline (PBS), fixed with 70 % ethanol over night at -20 °C, and intensively washed again. After treatment with 1 % RNase (Sigma) for 20 min at 37 °C, the DNA of the cells was labelled with propidium iodide (50 g/ml, Sigma) over night at 4 °C. Cells were measured in a FACSCalibur™ flow cytometer (BD Biosciences) equipped with a 488 nm argon-ion laser and a Macintosh Power PC (G4). In general, 25,000 events were acquired using CellQuest Pro 4.0.1. Proliferating cells (i.e. cells in S- and G2-phase of the cell cycle) and apoptotic cells (sub-G1 peak of the DNA histogram) were then calculated in percent using ModFIT version 3.0 (BD Biosciences).

### Adhesion of Living Lens Cells

Time dependent adhesion experiments with subconfluent and confluent living HLE-B3 cells were carried out using Bionas^®^ sensor chips for adhesion (Bionas GmbH, Rostock). The sensory component was impedance measurements with interdigitated electrode structures (IDES). The impedance and conductance (i.e. the ability of a material to pass electrons) correlates with the adhesion of cells [27,28]. The IDES sensor structure on the silicon chip consists of gold. The sensor chips were cleaned and sterilized with 70 % ethanol for 10 min and rinsed with DMEM before HLE-B3 cells were disseminated. For a subconfluent monolayer 8 x 10^4^ cells/chip were cultured for 24 h, and for a confluent monolayer 1.5 x 10^5^ cells/chip were cultured for 42 h in DMEM as describe above. Then, the cells grown on the chip surface were incubated with the substance Mibefradil at concentrations of 10, 20, 30 µM for 24 h and the physical parameters conductance and impedance were measured in the Bionas 2500 instrument (Bionas^®^ GmbH) with the software Bionas^®^ 2500 SW 1.15. There are no commercial relationships between Bionas^®^ GmbH and our department of cell biology.

### Statistical Analysis

Statistical analysis was performed with SPSS 14.0 for Windows (SPSS Inc., Chicago, IL). The differences between the concentrations were evaluated using Students t-test for independent samples because variables present normal distribution (Kolmogorov-Smirnov test). Data were indicated as mean and standard error of the mean. Significance was set at p < 0.05.

## RESULTS

In our new experiments we were interested in both, the time-dependent influence of Mibefradil on cell adhesion in subconfluent and confluent living cells *in vitro* and, we wanted to solve the question if this drug can be immobilized in PLGA micro particles which were then affixed at a capsular tension ring as a practicable approach.

**Fig. (3).**
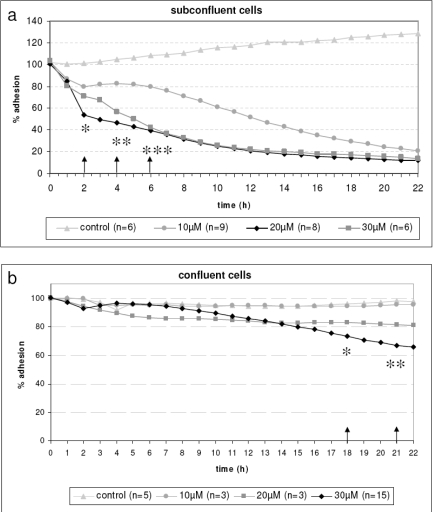
**a**: Time-dependent loss of adhesion of living subconfluent HLE-B3 lens epithelial cells on Bionas^®^ sensor chips. Note that in subconfluent cells, 20 and 30 µM Mibefradil induced a clearly decrease of cell’s adhesion capacity already after 2 h (* p<0.05, 4 h ** p<0.01, 6 h *** p<0.001) compared to the untreated control and, reached a nearly total inhibition after 20 h. Bionas 2500 instrument (Bionas^®^ GmbH). **b**: The influence of Mibefradil on confluent HLE-B3 lens epithelial cells is negligible. Here, only Mibefradil in the highest concentration (30 µM) inhibited cell adhesion first after 18 h (* p<0.05). The lower Mibefradil concentrations revealed no effect on living cell adhesion during the observed time frame.

For time-dependent adhesion analysis cells were grown on the sensor chips as seen in Fig. (**2**). The physical parameter impedance was measured with the adhesion sensor. This impedance signal correlates with the density of the cell monolayer during the experiment as well as with the adhesion behavior of the cells such as the spatial proximity of the cell membrane to the electrode and cell morphology like the cell-cell-connections (tight junctions). We revealed that the calcium channel blocker Mibefradil induced a loss of cell adhesion (Fig. **3a**). In subconfluent lens cells, we observed a clearly decrease at 30-40 % already after 2 h of Mibefradil incubation with 20 and 30 µM followed by a nearly total inhibition after 20 h demonstrating the loss of adhesion. This is in contrast to confluent cells (Fig. **3b**). In confluent cells, the cell monolayer is without any gaps in between the cells, therefore Mibefradil significantly influences the cell adhesion first at 18 h and only in the highest concentration of 30 µM. It is obviously, that Mibefradil’s influence is more intensive in proliferating lens epithelial cells (according to the subconfluent state in the cell culture).

**Fig. (4).**
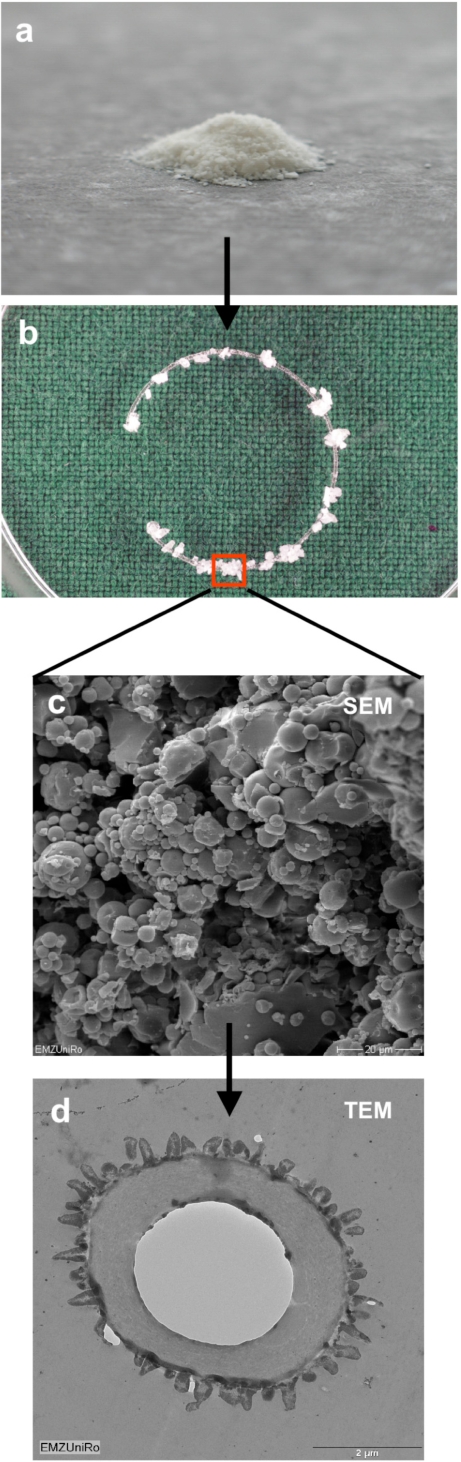
**a**: Spheric PLGA micro particles loaded with Mibefradil (Canon Powershot G6 Digicam, 4x optical zoom). **b**: Capsule tension ring with fixed spheric PLGA micro particles loaded with Mibefradil. The indicated area is seen in the next image below. **c**: Magnified view of the indicated area (in b) of the tension ring: scanning electron microscopic image of Mibefradil containing PLGA micro particles. d: Transmission electron microscopic image of Mibefradil containing PLGA micro particles.

The capsule tension ring with the affixed spherical PLGA micro particles which were loaded with Mibefradil is demonstrated in Fig. (**4**). The substructure of the micro particles is shown in the corresponding SEM- (Fig. **4c**) and TEM-images (Fig. **4d**). The emulsifying agent embeds the substance into small vesicles. Formation of PLGA and the form of spheric micro particles like in the TEM-image results from dehumidifying of the organic phase of the emulsifying agent. We wanted to know if Mibefradil incorporated in micro particles has the same cell inhibitory influence compared to the solved substance. In the cell analysis tests we can demonstrate that the release behavior of Mibefradil is effective with a daily average of 7.5 mg/ml of the active agent during a period of 50 days. More than 15 mg/ml of the substance was released into the medium during the first 24 h (personal communication J. Teller and [25]). The MTT analysis in Fig. (**5**) shows a reduction of the metabolic activity of HLE-B3 lens epithelial cells after the 24 h-incubation with Mibefradil-loaded particles (p<0.001). Flow cytometric measurements in Fig. (**6**) demonstrate that the synthesis phase (S) and the G2/M phase of the cell cycle of HLE-B3 lens cells are significantly reduced, and apoptosis in the late stage was induced due to Mibefradil immobilized in the

**Fig. (7).**
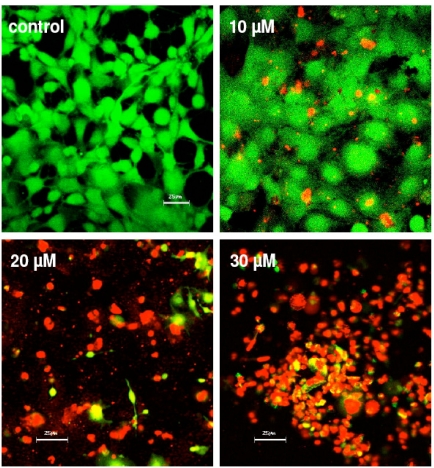
Live/dead viability test of HLE-B3 lens epithelial cells incubated with Mibefradil-containing micro particles after 24 h. Almost all cells of the control as well as of the Mibefradil concentration 10 µM seem to be alive (green), whereas incubation with 20 and 30 µM Mibefradil resulted in cell dead in these subconfluent growing cells (red).

**Fig. (8).**
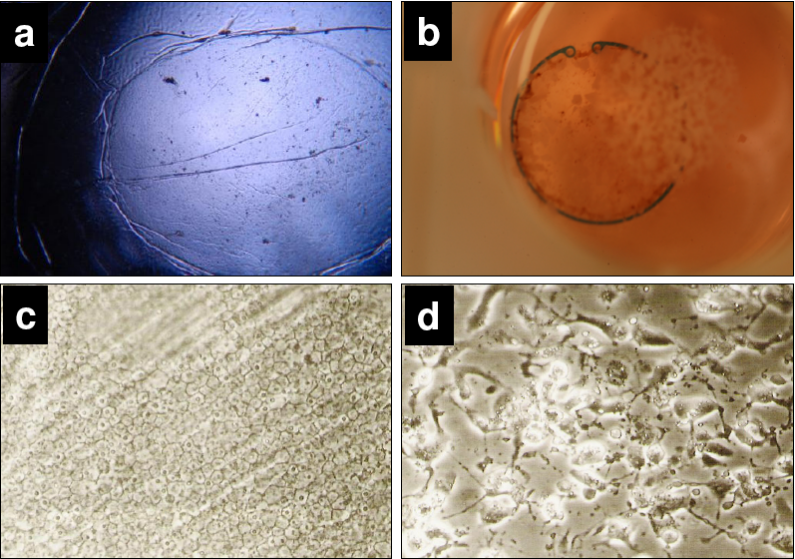
**a**: The capsular bag as a human organ culture model *in vitro*. **b**: Capsular bag with a normal capsule tension ring. **c**: Morphology of untreated, well spread human lens cells after 21 d in the organ culture model (microscopic images, 10x). **d**: Morphology of lens cells after treatment with Mibefradil-containing micro particles (30 µM) after 21 d in the organ culture model (microscopic images, 20x). Note that the cell area is reduced and neighbouring cells lost their cell-cell-contacts.

**Fig. (9).**
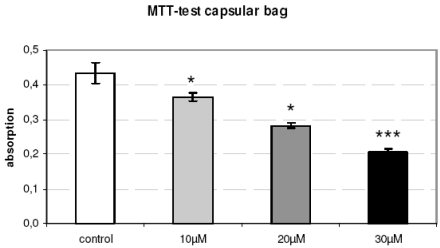
The metabolism of primary human lens epithelial cells in the organ culture model demonstrates a significant decrease after 21 d culture with a Mibefradil containing capsular tension ring at the range of 10, 20 and 30 µM (* p<0.05, *** p<0.001).

PLGA particles (20, 30 µM) (p<0.05). In addition, Mibefradil in release concentrations of 20 and 30 µM results in a massive cell disturbance and loss of vitality (Fig. **7**). In Fig. (**8**) we demonstrate the human capsular bag (Fig. **8a**) which was used as an organ culture model for the placement of the capsule tension ring (Fig. **8b**). After 21 days of cultivation time of the organ culture, in controls the cells grow out and reveal a well spread morphology (Fig. **8c**). In contrast, the morphology of Mibefradil (30 µM) treated lens cells in the capsular bag (Fig. **8d**) is completely different – the cell area is small and neighbouring cells lost their cell-cell-contacts. In addition, the metabolism of outgrowing primary human lens cells in the organ culture model (Fig. **9**) is significantly decreased after a 24 h-release (10, 20 µM: p<0.05 and 30 µM: p<0.001) of Mibefradil-containing micro particles which were fixed at the capsular tension ring.

## DISCUSSION

In our earlier investigations concerning the influence of the T-type calcium channel blocker Mibefradil in primary human lens epithelial cells in a monolayer cell culture we found out changes in the expression of integrin receptors [18] and the induction of apoptosis [16]. Reduced integrin expression leads to reduced adhesion as could be found e.g. in leucocytes [29,30]. Here, in our new experiments concerning time dependent cell adhesion with living human lens epithelial cells (HLE-B3) on adhesion sensor chips we revealed a reduced adhesion, e.g. the conductance and impedance values were diminished which is an indicator for the loss of cell’s contact area to the surface of the sensor. Interestingly, the concentration of the drug which effectively inhibits cell adhesion in subconfluent cells on the adhesion chips is not effective in confluent cells. One explanation for this important feature to inhibit only growing lens cells could be the role for T-type calcium channels in cell proliferation [31], as shown for example in fibroblasts [32] and in smooth muscle cells [33]. In these studies a pharmacological blockade of T-type channels completely inhibited proliferation and it could be found that the T-type voltage-operated calcium channels are required for the cell cycle progression and proliferation [33]. Otherwise, over-expression of a subunit of the T-channel led to a growth advantage in HEK-293 cells [34]. Our primary human lens epithelial cells as well as the lens epithelial cell line HLE-B3, both express calcium channels of the T-type, as we could demonstrated earlier for the first time [16,17]. Further experiments have to discover the expression differences of T-type channels in subconfluent versus confluent lens epithelial cells to finally conclude that the observed higher sensitivity to Mibefradil in subconfluent cells correlates with their calcium channel expression. To interfere only with the outgrowing, proliferating cells is an important feature for the protection of the intact collateral tissue in the eye [9] and offers possibilities in the specifically pharmacological intervention in the secondary cataract. Until now the use of pharmacological substances like cytostatic drugs and immunotoxins has been limited because of damages also of the surrounding tissues due to general toxic effects [35-37]. Mibefradil as T-channel inhibitor interfered especially with growing HLE-B3 cells expressing higher Ca_v_ 3.1 and 3.3 levels [17]. Therefore, the toxicity for the surrounding tissue is possibly not to expect in the therapeutic concentrations used for the prevention of lens epithelial outgrowth. In this context it is noteworthy that the substance Mibefradil, accredited in Germany 1997, was withdrawn from the market [38] because of interactions with the cytochrome p450 system of the liver. Mibefradil in combination with other pharmaceuticals in the antihypertensive therapy especially beta blockers, led to cross reactions and followed complications in patients. However, Mibefradil dihydro-chloride is interesting as T-channel blocker substance with a specific antiadhesive and antiproliferative properties different from existing calcium channel blockers such as Verapamil (L-type blocker) [17] or Ethosuximid (T-type blocker) (still unpublished data from contemporary experiments).

A capsular tension ring is used in cases undergoing intraocular lens implantation and it could be evaluated a safety and efficacy use associated with a reduced incidence of posterior capsular opacity [39]. We suggest that our pharmacologically modified capsular tension ring has the potential to prevent the migration of residual lens epithelial cells. Therefore, animal experiments have to evaluate the practicability of this new method to prevent postoperative cataract **in vivo**.

## CONCLUSION

The T-type-calcium channel blocker Mibefradil specifically interfere with outgrowing cells, which corresponds to a subconfluent cell culture. It is feasible to encapsulate the calcium channel blocker Mibefradil in PLGA micro particles, to immobilize the micro particles at the capsular tension ring consisting of PMMA and to get a release behavior of Mibefradil significantly effecting cell viability and growth. Our new approach using Mibefradil containing micro particles with a continuously released agent seems to be practicable to prevent cataracta secundaria.

## Figures and Tables

**Fig. (1):**
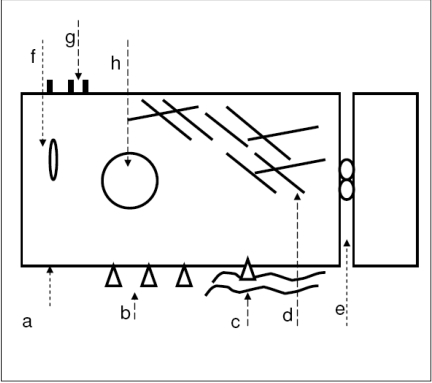
Schematic representation summarizing the effect of the T-calcium channel blocker Mibefradil on human lens epithelial cells found in our basic *in vitro* cell culture experiments: a. Membrane potential depolarized, b. Integrin expression reduced, integrins clustered, c. Extracellular matrix proteins clustered, d. Actin cytoskeleton fragmented, vimentin structure altered, e. Opening of the tight junctions − ZO-1 translocation to the cytoplasm, f. Apoptosis − Bax translocation to the mitochondria, g. Apoptosis − phosphatidylserine switch, h. Apoptosis − DNA cleavage.

**Fig. (2).**
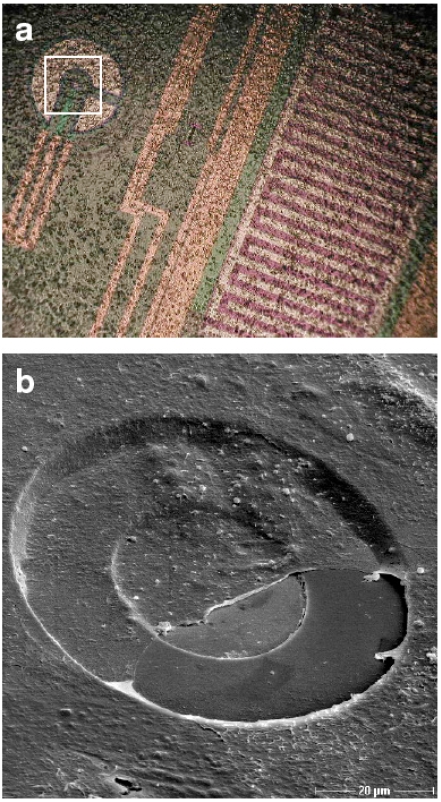
**a**: HLE-B3 lens epithelial cells grown on Bionas^®^ sensor chips for adhesion experiments in living cells (microscope Olympus BX51M, software Olympus DP-soft, camera Olympus Camedia C4040). The white insert is magnified in the SEM image below. **b**: Scanning electron microscopy of HLE-B3 on the sensor chip.

**Fig. (5).**
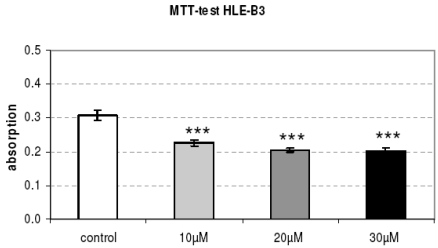
The metabolic activity (MTT test) of cultured HLE-B3 lens epithelial cells is significantly reduced after the 24 h-release of Mibefradil immobilized in micro particles (p<0.001).

**Fig. (6).**
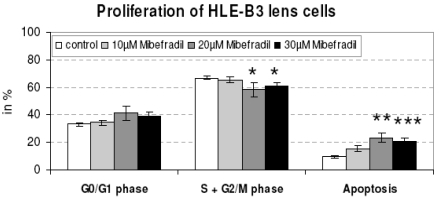
The synthesis phase (S) and the G2/M phase of the cell cycle of HLE-B3 lens epithelial cells is significantly reduced, and apoptosis in the late stage was induced after the 24 h-release of Mibefradil (20, 30 µM) immobilized in micro particles (n=6, * p<0.05, ** p<0.01, *** p<0.001). Flow cytometry.
